# Effect of Parental Age and Mating Status on Reproductive Performance of *Orius laevigatus* (Hemiptera: Anthocoridae)

**DOI:** 10.3390/insects13090827

**Published:** 2022-09-12

**Authors:** Amador Rodríguez-Gómez, Virginia Balanza, Alberto Donate, Ana Belén Abelaira, María del Carmen Reche, Isabel Sánchez-Martínez, Pablo Bielza

**Affiliations:** Departamento de Ingeniería Agronómica, Universidad Politécnica de Cartagena, 30203 Cartagena, Spain

**Keywords:** biological control agent, fecundity, *Orius laevigatus*, copulation, delayed mating, multiple mating, age

## Abstract

**Simple Summary:**

The successful function of biological control agents (BCAs) in a crop heavily depends on their reproductive potential, and it all begins at the mating. Reproductive potential is crucial for both the mass-rearing and field performance of natural enemies. The present study aimed to evaluate how many consecutive females of *Orius laevigatus* a male can mate and the effect on female’s reproductive performance. In addition, we wanted to see the effect of the age of the male and female at mating. In the multiple mating experiment, the males of *O. laevigatus* showed a high capacity to fertilize females successively, not reducing the fecundity until the sixth mated female. Therefore, the proportion of males in mass rearing could be reduced. In the delayed mating experiment, copulation duration and fecundity increased with the age of the male but decreased with the age of the female. In contrast, fertility (percentage of egg hatching) followed a pattern contrary to fecundity, increasing with female age but decreasing with male age, although it did not counterbalance the advantage granted by higher fecundity. In conclusion, reproductive capacity is increased with very young females and with males a few days old.

**Abstract:**

The reproductive potential of biological control agents (BCAs) is crucial for efficient mass-rearing and field performance, and it all begins with mating. Fecundity can be strongly influenced by intrinsic conditions, such as female age and, often neglected, male age and mating status. However, little is known about the impact of parental status at mating on female reproductive outcomes in BCAs. *Orius laevigatus* (Fieber) (Hemiptera: Anthocoridae) is widely used to control thrips in protected crops. We evaluated how many consecutive females a male could successfully mate and the effect on a female’s reproductive output. In addition, we studied the effects of male and female age on mating. In the multiple mating experiment, the males showed a high capacity to fertilize females successively, not reducing fecundity until the sixth mated female. In the delayed mating experiment, copulation duration and fecundity increased with male age but decreased with female age. In contrast, fertility followed an opposite pattern, increasing with female age but decreasing with male age. However, fecundity gains outweighed fertility declines in both sexes. Therefore, reproductive capacity is increased when mating newly emerged females with males a few days old. The implications of our results for mass rearing and field performance are discussed.

## 1. Introduction

Augmentative biological control is a very effective strategy for controlling pests, particularly in protected crops. However, despite its success, it is still underused due to a number of constraints that hinder wider adoption [1,2]. Many factors influence the effectiveness of biological control agents (BCAs), including reproductive performance. The successful establishment and multiplication of a BCA in a crop heavily depends on its reproductive potential, and it all begins at the mating for most species.

Fecundity has commonly been used as a criterion for quality control of BCAs [3] and as a proxy to estimate the response to diverse factors, such as prey and non-prey foods, rearing systems, body size and environmental conditions, among others [4,5,6,7], and potential fitness costs resulting from insecticide resistance [8,9,10]. However, female fecundity can be strongly influenced by intrinsic conditions, such as female age and, often neglected, male age and mating status. The role of males is usually overlooked when studying the reproductive performance of BCAs, but it may have a substantial effect [11,12]. Reproductive potential is crucial for both the mass-rearing and field performance of BCAs. Not surprisingly, it has been the focus of many studies aiming to enhance BCA commercial production and establishment in crops. However, little is known about the impact of parental status at mating on female reproductive outcomes.

*Orius laevigatus* (Fieber) (Hemiptera: Anthocoridae) is a key predator of thrips and other small pests and is widely used in greenhouse crops [13]. Male age at mating was found to have an impact on female reproductive potential under nutritional stress [12]. Females mated with newly emerged males (less than 24 h old = 0 days old) showed reduced oocyte counts compared to those mated with older males (8 days old) when fed on an artificial diet but not on an optimal food. However, further research is suggested to gain knowledge on the effect of older ages on the reproductive capacity of *O. laevigatus*. Surprisingly, to the best of our knowledge, no studies have been carried out on the effect of female delayed mating in this important BCA.

However, the *O. laevigatus* mating system has been studied by Leon-Beck and Coll [11]. Females were found to be monandrous and males polygamous, successfully mating with up to three virgin females sequentially offered. However, fecundity decreased in the third female mated, which was attributed to sperm depletion in twice-mated males, but more investigation is needed to determine the number of females a male can successfully mate.

Both factors, the male’s mating history and the age of the male and female at mating, can have a significant impact on the biotic potential of this predator, having implications for its industrial mass production and its biocontrol function in crops. Therefore, the present study aimed to evaluate how many consecutive females a male can successfully mate and the effect on a female’s reproductive output. In addition, we wanted to see the effect of the age of the male and female at mating.

## 2. Materials and Methods

### 2.1. Insects

Thirty-five wild populations of *O. laevigatus* were collected in different areas of Spain and Mediterranean countries between 2012 and 2015 (see [8,9,10] for details). Individuals were collected from wild plant species in natural habitats. A reference wild population (WildMix) originated from a mixture of all the wild populations collected and was founded and reared over 10 generations prior to experiments. Populations were reared using 1-liter plastic containers with filter paper on the lid. *Ephestia kuehniella* Zeller (Lepidoptera: Pyralidae) eggs were used as food and green bean (*Phaseolus vulgaris*) pods were used as a source of moisture and oviposition substrate. Containers were kept at 26 ± 1 °C, 65 ± 5% R.H. and a 16:8 (L:D) h photoperiod.

### 2.2. Experiments

The experiments were carried out with female and male adults from the reference population (WildMix). In the multiple mating experiment, to study the effect of the mating status of the male, the last instar nymphs were placed individually in 5 mL vials with food to prevent mating upon adult emergence. Newly emerged adults (<24 h) were sexed and used in the experiment. To allow mating, a male and a female were simultaneously introduced into a 5 mL vial, where the pre-mating and mating times were recorded. Once the male was uncoupled, the female was withdrawn and a 10-min resting period was allowed. After the resting period, a new virgin female was introduced. Each male was mated with six consecutive females, totaling 23 males with their respective six females (138 females).

In the delayed mating experiment, to investigate the effect of the age of the male and female at mating, the last instar nymphs were isolated at different times to obtain virgin adults of different ages (0, 7 and 14 days). On the one hand, to study the effect of female age, newly emerged males (M0) were coupled with females of one (F7) or two weeks (F14) of age. On the other hand, to study the effect of male age, newly emerged females (F0) were mated with males one (M7) or two weeks (M14) old. For each cross, 30–40 couples were used. As in the multiple mating experiment, a male and a female were placed in the same 5 mL vial, where the pre-mating and mating durations were timed. Both the multiple and delayed mating experiments were carried out at the same time; therefore, data for newly emerged males (M0) crossed with newly emerged females (F0) were considered from the first experiment, including only the data for the first females mated (both virgin adults).

For both experiments, after each mating, the females were individually deposited in 30 mL plastic cups covered with paper to allow ventilation containing *E. kuehniella* eggs ad libitum and a piece of green bean pod. The females were kept at standard laboratory conditions, 26 ± 1 °C, 65 ± 5% R.H. and a 16:8 (L:D) h. Every 2–3 days, food was added, and the bean was exchanged for a fresh piece until female death. Longevity, early fecundity (10 days) and lifetime fecundity were assessed. Fertility (proportion of eggs hatched) was calculated with the eggs laid for the first 10 days.

### 2.3. Data Analysis

In the multiple mating experiment, data were analysed using a one-way analysis of variance, with the order of the female mated (one to six) as the main factor. In the delayed mating experiment, a one-way ANOVA was used with age (0, 7 and 14 days) as the main factor within each sex. Assumptions of normality and homogeneity of variances were checked prior to the analysis. We transformed proportions into their arcsine values if assumptions of normality and homogeneity were not met. When significant differences between populations were observed, the means were separated using Tukey’s HSD test. Females who didn’t lay eggs were excluded from the analysis.

## 3. Results

### 3.1. Multiple Mating Experiment

The mating status of the male affected the pre-mating time, with significant differences (F = 2.58, df = 5/109, *p* < 0.05), showing longer times when mating the last females (Table 1). On the other hand, there were significant differences (F = 21.39, df = 5/109, *p* < 0.001) in the copulation duration (Table 1). The male took longer to copulate the first female, drastically reducing that time with the second female, with a progressive reduction with the subsequent females. From the results obtained from all the females, the minimum mating time for insemination to be effective (resulting in oviposition) was 61 s.

**Figure 1:**
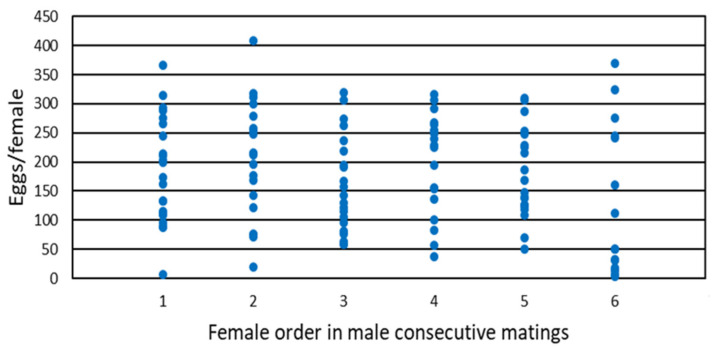
Lifetime fecundity of individual females of *Orius laevigatus* mated in sequential order by a male.

No significant differences among females were observed in their lifetime fecundity (F = 1.64, df = 5/109, *p* > 0.05) (Table 1). However, a trend toward lower fecundity was observed in the sixth female. Figure 1 represents the total fecundity of each individual female according to mating order. There is little variation in fecundity among females depending on which order was copulated by the male. However, there was much variation among the individual females within each order. For example, females mated first (by a virgin male) had a fecundity of 7 to 366 eggs/female, and females mated second from 19 to 409 eggs/female. Despite this high variation, it is appreciated that for females mated from first to fifth place, few females laid below 50 eggs (one or none), but there were up to 8 females with low oviposition in the females mated in sixth place.

Indeed, in early fecundity (10 days), significant differences were observed (F = 5.39, df = 5/109, *p* < 0.001), with significantly lower fecundity in females mated in sixth place and the fifth females in an intermediate position (Table 1).

The last females that the male mated, fifth and sixth especially, tended to have higher longevity than the females previously mated (1st, 2nd and 3rd) (F = 2.66, df = 5/109, *p* < 0.05) (Table 1). The female order had no effect on the percentage of egg hatching (F = 1.07, df = 5/109, *p* > 0.05) (Table 1).

### 3.2. Delayed Mating

The time required for pre-mating varied significantly between females with different ages (F = 4.69, df = 2/123, *p* < 0.05), but not between males (F = 1.57, df = 2/123, *p* > 0.05) (Table 2 and Table 3). When the females were newly emerged, the pre-mating time was much shorter than when they were 7 or 14 days old (Table 2).

In contrast, the time for copulation was significantly shorter (F = 20.82, df = 2/123, *p* < 0.01) for older females than for newly emerged females (Table 2). On the contrary, the mating time was significantly longer in older males (F = 19.70, df = 2/123, *p* < 0.01) (Table 3).

Only marginal significant differences were detected (F = 2.75, df = 2/123, *p* = 0.07) in total fecundity as a function of the age of the female, but there were as a function of the age of the male (F = 3.18, df = 2/123, *p* < 0.05) (Table 2 and Table 3). However, for early fecundity (10 days), young females laid significantly (F = 7.25, df = 2/123, *p* < 0.05) more eggs than one- or two-week-old females (Table 2). There was also a significant effect of male age (F = 6.79, df = 2/123, *p* < 0.005) in early fecundity, having the females mated by the young males lower early fecundity than those females mated with older males (Table 3).

Either female (F = 4.19, df = 2/123, *p* < 0.05) or male age (F = 4.00, df = 2/123, *p* < 0.05) significantly affected egg hatching. Fertility increased with the age of the female but decreased with the age of the male (Table 2 and Table 3).

The longevity of the females also varied significantly depending on the age of the female (F = 5.57, df = 2/123, *p* < 0.05) and of the male (F = 4.78, df = 2/123, *p* < 0.05). Females who were fertilized at an older age lived longer than those who were fertilized when they had just emerged (Table 2). Unexpectedly, the age of the male also seemed to influence the longevity of the females, with females mated to older males showing a shorter life span (Table 3).

## 4. Discussion

### 4.1. Multiple Mating

Our results show that the minimum mating time for effective insemination was 61 s. In that case, the total fecundity was low (33 eggs), but in another female with a mating time of 63 s, the total fecundity was already considerable (126 eggs). These results differ from those obtained in another study that set this minimum time at 105 s for *O. laevigatus* [11]. This difference could be attributed to the use of different populations. As discussed later, our population seems to show a higher vigor, which could also explain this fact. For other *Orius* species, longer minimum mating durations have been reported: 90 s for *O. insidiosus* (Say) [14], 124 s for *O. minutus* (L.), 219 s for *O. sauteri* (Poppius), and 273 s for *O. strigicollis* (Poppius) [15].

Copulation duration decreased from the first female mated. For the first female, the copulation duration was significantly longer (270 s) than for the second (184 s) and successive females (116–166 s). Several factors may be involved in this phenomenon. One might be sperm depletion after consecutive matings. The reduction in copulation duration in sequential matings of *O. laevigatus* was observed by Leon-Beck and Coll [11] when copulations were successive on the same day, but not with a 2-day resting period elapsed between copulations. Sperm depletion might also be associated with a reduction in fecundity. In our study, the number of eggs laid only decreased in the sixth female mated and marginally in the fifth one. The quantity of ejaculate passed to females may be in excess to fully fertilize a female, and thus sperm depletion may not have an impact on fecundity until a minimum threshold is reached. In the seed beetle *Callosobruchus maculatus* (Fabricius) (Coleoptera: Chrysomelidae), despite the decline in ejaculate volume from the first mating, fecundity was not reduced until the fourth mating.

Leon-Beck and Coll [11] attributed the reduction in fecundity in the third female mated by the same male regardless of the refractory time between male copulations (0, 1 and 2 days) to sperm depletion. However, when matings took place on the same day, copulation duration was significantly longer with the first female mated than with subsequent females, but when two days were left between matings, the mating duration was similar for the three females. Thus, the duration of copulation is not directly related to the amount of sperm available in the male and seems to be attributable to the energetic exhaustion of the male. With the first female, the male is able to maintain a longer copulation, but this is gradually reduced in successive copulations. Copulation requires great effort for the male, as it has to overcome the initial resistance of the female.

There were no significant differences in total fecundity depending on mating order, although a decrease was observed in the sixth female (Table 1). As mentioned above, Leon-Beck and Coll [11] did observe a significant reduction in total fecundity already in the third female, concluding that there was a depletion of sperm after three consecutive copulations, regardless of whether they were on the same day, or 1 or 2 days were left between them. However, in our study, this quick decrease in available sperm was not observed, since even some females mated in sixth place in a consecutive way were capable of laying many eggs. In fact, the second (369 eggs) and fourth (324 eggs) most fecund females were fertilized in sixth place. However, more females showing low fecundity were found in females mated in sixth place. Eight out of the eleven least fecund females (<51 eggs) had been fertilized in sixth place. Therefore, males are capable of maintaining their insemination capacity at high levels after six copulations in a row, but generally, it tends to decrease. In other *Orius* species, namely *O. minutus*, *O. sauteri* and *O. strigicollis*, the number of eggs laid was not reduced after consecutive matings of the same male with three females [15].

The lack of significant differences in total fecundity may be due to the high variability due to other factors. In fact, it is typical in this species to find females laying a high number of eggs, while others, from the same population and reared under the same conditions, lay significantly fewer eggs. For example, it has been reported that longevity has a significant influence on total fecundity, since longer-lived females have a greater opportunity to lay eggs [7,12]. Therefore, the measurement of early fecundity has been proposed as a better indicator of the effect of external factors on the fecundity of females [7], yet it is a good indicator of expected total fertility. There were significant differences in 10-day fecundity depending on the mating order, showing the sixth female a lower early fecundity than the previous ones, with the fifth female in an intermediate position. Thus, the insemination capacity of *O. laevigatus* males remained without reduction, at least until the fourth consecutive fertilized female. This contrasts with the results of Leon-Beck and Coll [11], who concluded that it had already decreased in the third female. This difference could be explained by the different populations used, ours being a mixture of a wide range of wild populations compared to the commercial population used in the aforementioned work. Our population seems to show greater vigor, measured both by a greater insemination capacity of the male, as has been commented, as well as by a longer average longevity (51.0 days versus 27.0 days), a greater total fecundity (186 eggs/female on average for the first female compared to around 90 eggs/female), as well as a shorter effective copulation duration (61 s vs. 105 s), as mentioned above.

All this leads us to reflect on the convenience of using more than one population of a species when studying its biological and ecological characteristics. In *O. laevigatus*, this point has already been highlighted by showing the high variability between populations in characters as diverse as their susceptibility to insecticides [8,9,10], their body size [7], their adaptation to alternative food such as pollen [6] or their predation capacity [16].

### 4.2. Delayed Mating

The shortest copulation duration resulting in oviposition was 58 s, confirming the minimum mating time (61 s) found in the former experiment, both significantly shorter than previously reported for *O. laevigatus* and other *Orius* species [11,15,16].

Copulation duration increased with the age of the male. Newly emerged males (<24 h) may not have fully matured and thus not perfectly ready for mating. In fact, in other *Orius* species, it has been described that males emerge before females to be ready for mating [17,18]. With the age of the male, the copulation duration is possibly increased by a larger quantity of sperm stored and then transferred.

In contrast, copulation duration decreased with the age of the female. Males of some species have been observed to transfer more sperm to young females than to older females [19]. In this way, males might transfer less sperm when mating with older females, and therefore, the copulation duration decreases. This is also supported by the lower fecundity of older females.

The age at mating affected lifetime and early fecundity but in opposite ways for males and females. The females mated with the oldest males (7 and 14 days old) laid more eggs than those fertilized by recently emerged males. In contrast, young females were more fecund than older females.

Indeed, fecundity increased with the age of the male and decreased with the age of the female. The fact that females mated with older males were more fecund also suggests that older males have completed sexual maturation and have a larger amount of accumulated sperm, leading, in turn, to increased copulation duration. These results are consistent with other studies in other species, where young males had less sperm [20,21]. Therefore, older *O. laevigatus* males may have more sperm available than younger ones, favoring the number of eggs laid by females. Our results are similar to those of Bonte and De Clercq [12] with *O. laevigatus*. Females fertilized with 8-day-old males had higher early fecundity than those mated with newly emerged males. When fed a nutritionally rich diet, such as *E. kuehniella* eggs, no significant differences were detected, but females mated by young males had a fecundity of almost half (38 eggs) of that of females mated by 8-day-old males (71 eggs). In addition, with a more restrictive diet, such as an artificial diet, significant differences were observed, with higher fecundity in females mated by 8-day-old males compared to those mated by newly emerged ones. Our results suggest that with age (1 or 2 weeks), males increase the amount of sperm, providing more viable sperm to the female and therefore improving its fecundity. As mentioned above, in some *Orius* species, males develop faster than females, especially at low temperatures, emerging earlier and then preparing for mating [17,18]. According to our results, this temperature-regulated protandry [22] confers an ecological advantage by increasing female fecundity.

In the case of females, there were also differences in fecundity between ages, with young females showing higher fecundity than females 7 and 14 days old. In many species of insects, this same phenomenon has been reported; females mated at young ages are more fecund than those in which mating is delayed. This effect has been explained by the deterioration or reabsorption of maturing oocytes [23,24].

Fertility (percentage of egg hatching) followed a pattern contrary to fecundity, increasing significantly with the age of the female but decreasing with the age of the male. In a previous work with *O. laevigatus*, fertility did not vary significantly with the age of the male, with a difference of 8 days [12]. In our case, there were also no significant differences between the newly emerged males and those 7 days old, but there were with those 14 days old. These results suggest that although the amount of sperm may increase with the age of the male, resulting in higher fecundity, sperm quality seems, in turn, to decline, thus reducing fertility. The effect of male age on sperm quality has been found in previous studies on other insect species [25,26]. Age-specific reduction in fertilization success may be due, on the one hand, to DNA damage or deterioration of the spermatozoan cell membrane in stored sperm or, on the other hand, to negative effects of male age on spermatogenesis [27].

The opposite occurs in females; as the age at mating increases, fecundity declines, but fertility increases. In other species, it has been observed that both the fecundity and fertility of the female decrease with the age at mating [23]. We have not found a clear explanation for our results with *O. laevigatus*. Perhaps it influences the family Anthocoridae, together with Cimicidae, to have a very different fertilization system from the rest of the insects [24].

Despite the opposite effect of age on fecundity and fertility in both males and females, the overall effect on reproduction is that of fecundity. The decline in fertility did not counterweight the increase in fecundity in both males and females. Therefore, the biotic potential (nymphs hatched per female) increased with male age and decreased with female age. Optimal benefits for improved reproduction will result from young females mating with males 7-days old.

Females with delayed mating (7 and 14 d) lived longer than those mated newly emerged, suggesting that the time before mating and then without laying eggs did not involve significant energy depletion. This is in good agreement with the fact that virgin females have greater longevity than lay eggs [11]. 

The age of the male also appeared to influence the longevity of the females, with females fertilized by older males being less long-lived. This can be explained because the fecundity of the females increased with the age of the male, which resulted in higher energy expenditure that led to lower longevity. The development of eggs in the oviposition process requires high energy costs [28,29].

## 5. Conclusions

According to our results, males of *O. laevigatus* have a high capacity to fertilize females successively, not reducing fecundity until the sixth mated female. Therefore, although the sex ratio of the species is close to 1:1, this high proportion of males is not necessary for the mass rearing of this natural enemy. Thus, it would be interesting to develop a methodology to reduce the proportion of males since they consume food resources when not so many are needed.

On the other hand, it has been seen that the fecundity of the female is higher when the male is not newly emerging at mating and when the female is newly emerging. Although fertility varies in the opposite direction, it does not counterbalance the advantages granted by higher fecundity. Thus, reproductive capacity is increased in very young females and in males a few days old. Therefore, it would be interesting to add older males to rearing systems when adults are about to emerge, as also suggested by Leon-Beck and Coll [11] and Bonte and De Clercq [12]. Thus, a high proportion of newly emerged females will mate with older males. However, in this way, the number of males would greatly increase, consuming more food resources. Therefore, it would be very convenient to develop the proposed method of extracting males from rearing systems.

## Figures and Tables

**Table 1:** Effect of female order on mating time, fecundity, fertility and longevity of *Orius laevigatus* females mated successively with the same male.

Female Order	Pre-Mating Time (s)	Mating Time (s)	Total Fecundity (Eggs/Female)	First 10-Day Fecundity (Eggs/Female)	Fertility (Eggs Hatched)	Female Longevity (Days)
1	4.8 ± 0.6 b	270.0 ± 9.9 a	185.9 ± 19.4 a	58.4 ± 3.4 ab	0.90 ±0.01 a	47.± 5.3 bc
2	9.4 ± 3.7 b	184.3 ± 12.6 b	202.6 ± 23.3 a	65.2 ± 4.2 a	0.89 ± 0.02 a	47.7 ± 5.0 bc
3	20.0 ± 4.9 b	165.6 ± 12.7 bc	160.7 ± 18.1 a	59.0 ± 2.5 ab	0.88 ± 0.02 a	38.9 ± 4.7 c
4	28.4 ± 17.9 b	142.9 ± 12.9 cd	203.2 ± 18.7 a	60.5 ± 3.7 ab	0.85 ± 0.03 a	51.0 ± 6.2 abc
5	67.8 ± 32.3 a	130.9 ± 13.5 d	178.8 ± 17.1 a	50.9 ± 2.7 b	0.89 ± 0.02 a	57.6 ± 5.5 ab
6	54.1 ± 25.5 ab	115.7 ± 9.9 d	128.9 ± 33.2 a	39.1 ± 6.2 c	0.91 ± 0.02 a	64.2 ± 6.8 a

Means ± SE within a column followed by the same letter are not significantly different (*p* > 0.05; Tukey test).

**Table 2:** Effect of female age at mating on mating time, fecundity, fertility and longevity of *Orius laevigatus* females.

Female Age (Days)	Pre-Mating Time (s)	Mating Time (s)	Total Fecundity (Eggs/Female)	First 10-Day Fecundity (Eggs/Female)	Fertility (Eggs Hatched)	Female Longevity (Days)
0	4.8 ± 0.6 b	270.0 ± 9.9 a	185.9 ± 19.4 a	58.4 ± 3.4 a	0.90 ± 0.01 b	47.1 ± 5.3 b
7	21.8 ± 7.6 a	184.7 ± 18.0 b	156.4 ± 17.5 a	46.3 ± 4.3 b	0.91 ± 0.02 ab	59.8 ± 4.4 a
14	16.6 ± 4.3 a	198.6 ± 22.2 b	175.8 ± 16.1 a	50.3 ± 3.3 b	0.93 ± 0.01 a	60.8 ± 3.8 a

Means ± SE within a column followed by the same letter are not significantly different (*p* > 0.05; Tukey test).

**Table 3:** Effect of male age at mating on mating time, fecundity, fertility and longevity of *O. laevigatus* females.

Male Age (Days)	Pre-Mating Time (s)	Mating Time (s)	Total Fecundity (Eggs/Female)	First 10-Day Fecundity (Eggs/Female)	Fertility (Eggs Hatched)	Female Longevity (Days)
0	4.8 ± 0.6 a	270.0 ± 9.9 b	185.9 ± 19.4 b	58.4 ± 3.4 b	0.90 ± 0.01 a	47.1 ± 5.3 a
7	7.4 ± 1.3 a	304.3 ± 12.8 a	222.1 ± 12.6 a	60.8 ± 2.0 a	0.89 ± 0.01 ab	43.9 ± 4.0 ab
14	9.5 ± 2.5 a	354.5 ± 27.4 a	202.3 ± 27.4 ab	68.6 ± 6.6 a	0.86 ± 0.03 b	39.8 ± 5.2 b

Means ± SE within a column followed by the same letter are not significantly different (*p* > 0.05; Tukey test).

## Data Availability

The datasets generated and/or analyzed during the current study are available from the corresponding author on reasonable request.
